# Supplementary material for: Genomic characterization of the HER2-enriched intrinsic molecular subtype in primary ER-positive HER2-negative breast cancer
Source: Nat Commun. 2025 Mar 5;16:2208. doi: 10.1038/s41467-025-57419-z (PMC11882987; doi:10.1038/s41467-025-57419-z)
Supplement: Supplementary file 1 — Supplementary Information [file 41467_2025_57419_MOESM1_ESM.pdf]

SUPPLEMENTARY METHODS

Analysis of clinicopathological and molecular subtype variables

The clinicopathological characterization of HER2E breast cancer included analyses to determine differences of the clinical variables between luminal breast cancers belonging to the PAM50 subtypes LumA, LumB, and HER2E. Fisher's exact tests were employed to compare the differences in tumor grades, lymph node statuses, and HER2-low frequency. The clinicopathological variables age and tumor size were assessed using either a two-sided Student's or Welch's t-test. All reported p-values from statistical tests are two-sided.

SCAN-B samples were classified into PAM50 subtypes using the extended Nearest Centroid (NCN) approach, as described and performed by Staaf et al. This method extends the traditional nearest centroid classification by utilizing multiple reference sets for normalization, rather than a single set (see [1] for further details on NCN). The final PAM50 subtype for each sample was determined by a majority vote across 100 iterations of the classification process, while PAM50 subtype correlations used in the main manuscript Figure 3 K-L were calculated as the mean of all computed correlations (n=100) in the NCN approach.

21-gene Recurrence Scores for LumA, LumB and HER2E ERpHER2n tumors were calculated based on RNA-sequencing FPKM data using the Genefu R package (v2.36.0) and classified as either high, intermediate or low-risk cases [2]. The ROR scores and risk groups were previously calculated by Staaf et al. [1] and are provided in Supplementary Table 1 of that study. Specifically, we utilized the "NCN.ROR.risk.cat" column to obtain ROR risk groups. The ROR score was derived from the "NCN.ROR.asT0 column" for tumors ≤20 mm, and from the "NCN.ROR.asT1" column for tumors >20 mm. Tumor size information was also provided as column "Size.mm" in the supplementary file. Statistical significance of differences in 21-gene Recurrence Scores, ROR scores, and risk classifications were assessed using Mann-Whitney U and Fisher's exact tests, respectively. All reported p-values from statistical tests are two-sided.

Mutational Signature analysis

Mutational enrichment analysis was performed to identify potential mutational processes and driver alterations specific to the HER2E subtype compared to LumA and LumB tumors. Analyses in the SCAN-B cohort were based on data generated by WGS (HER2E n=28) and complemented by WGS data from the BASIS cohort using PAM50 subtype calls from the original BASIS study (LumA n=73; LumB n=105). BASIS mutational data was obtained as pre-processed and filtered data from a repository associated with the original study. For each SCAN-B patient, a tumor sample taken at surgery, and matched blood DNA was sequenced at Novogene (Novogene, UK) using 150bp paired end sequencing on an Illumina Novaseq to reach 120-150GB of sequence, resulting in a tumor coverage between 26-50X (median 34X) after duplicate removal and final filtering. Resulting BAM files were aligned to the reference human genome (GRCh38) using dockstore-cgpmap v3.2.0 implementing bwa mem 0.7.17-r1188 https://quay.io/repository/wtsicgp/dockstore-cgpmap. Mutation calling of WGS data for SCAN-B tumors was performed as described previously [3]. Briefly, the mutation calling pipeline was containerized within dockstore-cgpwgs v2.1.1 (https://quay.io/repository/wtsicgp/dockstore-cgpwgs) , implementing Caveman 1.13.15 for somatic substitution calling (Cancer Variants through Expectation Maximization: http://cancerit.github.io/CaVEMan/), Pindel 3.2.0 for detection of somatic small insertions and deletions (http://cancerit.github.io/cgpPindel/), and BRASS for structural rearrangements (BReakpoint AnalySiS; https://github.com/cancerit/BRASS). ASCAT 4.2.1 (https://github.com/cancerit/ascatNgs) was used to supply average ploidy and purity inputs for Caveman. Additional filtering was applied as follows; single base substitutions (SBSs) were filtered based on a PASS criterion, CLPM=0.00, and ASMD>=140, small indels were filtered by QUAL>=250 & REP<10, and structural variants filtered for BRASS assembly score >0 indicating successful de novo local assembly using Velvet to determine exact coordinates and features of breakpoint junction sequence.

Mutational signatures were assigned using Fit Multi-Step (FitMS) [4] (https://github.com/Nik-Zainal-Group/signature.tools.lib) and the following parameters were used. For substitution signatures, reference SBS previously identified in breast cancer were used in combination with high confidence rare signatures from any organ. A fixed threshold of 5% of the total

mutations contributing to the signature was applied before assigning a signature and an error reduction of 20% used. For structural rearrangement signatures, reference signatures previously identified in breast cancer were fitted to all samples with a total number of rearrangements greater than 25. In addition, mutational signatures were only assigned if a minimum of 5 variants and a minimum percentage of 5% of the total rearrangements were contributing to the signature. The resulting signatures were used as input to HRDetect [5].

Driver mutation analysis

Somatic mutations were annotated to Ensembl v91 for using (https://github.com/cancerit/VAGrENT). Non-synonymous point mutations and small indels were assessed for potential driver mutations by comparison to the list of genes in the Cancer Gene Census (https://cancer.sanger.ac.uk/census) and gene previously identified as driver genes breast cancer in a previous study [3]. Mutations within these genes were considered to be potential drivers if the same mutation exists multiple times in the COSMIC database or were reported as pathogenic or likely pathogenic in cancer in ClinVar. In addition, mutations in genes which are reported in the Cancer Gene Census as tumor suppressor genes (excluding those genes where the know mechanism of mutation was restricted to gene fusions) were considered to be potential drivers if the mutation was predicted to result in a premature truncation (nonsense, essential splice, frameshift mutations). Loss of all wild type alleles in tumor suppressor genes due to loss of heterozygosity was assessed using the ASCAT copy number for the corresponding segment.

In the SCAN-B/BASIS cohort we compared mutational and rearrangement signatures, as well as the overall tumor mutational burden between PAM50 subtypes and used Mann-Whitney U tests to assess statistical significance. All reported p-values from statistical tests are two-sided. The mutational calls for METABRIC were originally generated by a targeted NGS DNA-based panel [6, 7] and were obtained as ready to use calls. For both METABRIC and SCAN-B/BASIS we visualized the mutational landscape of SBSs, indel, and copy number driver genes within the HER2E subtype by creating waterfall plots using the GenVisR package (v1.34.0) [8]. The statistical significance of differences in *ERBB2*, *ESR1*, *TP53*, *PIK3CA*, and *FGFR4*

mutational frequencies was assessed using Fisher's exact tests. All reported p-values from statistical tests are two-sided.

Copy number alteration analyses

Copy number analyses were performed in the METABRIC cohort using Affymetrix SNP6 array data (n= 999 cases in total of which n=58 classified as HER2E) and for SCAN-B (n=28 HER2E cases) using copy number estimates obtained from WGS. For WGS-based SCAN-B data we compared HER2E profiles to copy number estimates from ERpHER2n LumA (n=73) and LumB (n=105) tumors from the BASIS cohort. WGS data was processed for copy number analysis by a modified ASCAT v3.1.2 version. Necessary reference files for this version are available at https://github.com/VanLoo-lab/ascat/tree/master/ReferenceFiles/WGS. Changes to the ASCAT algorithm are outlined at https://github.com/nnordborg/ascat/tree/scanb ("Changes in the SCANB-fork"). Baseline parameters were imbalance.test=bimodality_coefficient and tau=0.4. ASCAT segments were called as copy number gain or loss considering the tumor ploidy as described by Staaf et al. [9]. To compare alterations of genomic positions between SCAN-B and BASIS we mapped ASCAT segments to genes coordinates from the respective genome build (hg19 for BASIS and hg38 for SCAN-B) using the GenomicRanges (v1.50.2) R package [10]. In METABRIC, deposited copy number data for 22544 genes provided a copy number state of neutral (0), gain (1), amplification (2), loss (-1), or low-deletion/homozygous deletion (-2). In both SCAN-B and METABRIC, we summed up all copy number alteration for each tumor and subsequently compared the fraction of altered genomes between the PAM50 subtypes using Mann Whitney U tests. We tested all genes for significant differences in gain or loss frequencies between subtypes using Fisher's exact tests and corrected for multiple testing using FDR. For the significantly differing genes we then calculated the absolute differences in alteration frequency and visualized them using boxplots. Spatial copy number alteration profiles were plotted, showing the frequency of gain and loss alterations for HER2E, LumA, and LumB subtypes across the whole genome. All reported p-values from statistical tests are two-sided.

Gene network analyses

Gene network analyses of HER2E DEG genes were performed as outlined by Fredlund et al. [11] using SCAN-B FPKM data and visualized using Cytoscape [12]. Briefly, to identify coexpressed genes in the network analysis described by Fredlund et al. we used Spearman correlation with a cut-off of 0.5 in correlation to identify coexpressed genes, and with a requirement of at least four connections. Connections were outputted as a sif file and loaded into Cytoscape (v3.9.1) where two networks were identified and saved as gene lists for pathway analysis and transcription factor binding site analysis. Pathway analysis was performed similarly as for differential gene expression analyses previously outlined in the study.

Transcription factor binding site analysis

In a previous study it was demonstrated that genes co-expressed might be co-regulated by transcription factors (TFs) [13]. To explore common or enriched co-regulators, i.e., TFs, in our data we used the SMART software [14]. This software utilizes transcription factor binding sites (TFBS) represented as position weight matrices (PWM). These matrices describe the probability distribution of the four possible nucleotides at each location in the TFBS. They are derived from TRANSFAC v2020.1 [15] and JASPAR v2020 [16] databases, serving as surrogates for TFs.

The SMART TFBS analysis workflow starts by downloading promoter sequences from the UCSC genome browser database using the "refGene" coordinates. Specifically, a 1500bp upstream and 500bp downstream genomic interval relative to the most distal RefSeq gene transcription start site (TSS) is considered. TFBSs are mapped using PWMs on the downloaded promoter sequences, and binding quality scores are calculated, including a Core Similarity Score (CSS) for the first five most conserved consecutive positions and a Matrix Similarity Score (MSS) considering all positions of the matrix. To identify potential TFBS, SMART uses CSS threshold values of 1.0 and MSS threshold values of 0.9. Subsequently, a Genome-wide Transcription Factor Binding Sites (GTBS) database is created, containing information about gene promoter sequences and each TFBS's genomic positions and binding quality scores. In this study, UCSC genome build hg38 and 24861 gene promoter sequences were used to build two GTBS using PWMs from the TRANSFAC and JASPAR databases. Using the above build

database, SMART extracts TFBS information such as genomic positions and quality scores for the query genelist assumed to be coexpressed. Due to their small size in length and similar with differences of few bases, these binding sites can be detected extensively across the genome. To determine significant enrichment or common TFBS, SMART compares the mapping results of the query genelist with 100,000 randomly selected independent genelists of the same length from the database.

SMART generates two significance p-values for each TFBS:

1. Significant Enrichment (P_E): The probability of finding the total count number of a TFBS (C_T) from the query genelist (QG) being greater than or equal to that in a randomly selected genelist analyses (RSG) of the same length as the query list.

$P_E\ (\mathrm{x}) = \frac{\text{number of RSG having } C_{Tx} >= C_{Tx} \text{ of QG}}{100000}$, where x is a transcription factor binding site. (Eq 1)

2. Significant Presence (P_P): The probability of finding the observed number of promoters with at least one instance of the TFBS (CP_T) in a randomly selected genelist analyses of the same length as the query list.

$P_P\ (\mathrm{x}) = \frac{\text{number of RSG having } CP_{Tx} >= CP_{Tx} \text{ of QG}}{100000}$, where x is a transcription factor binding site. (Eq 2)

Both probability values P_E and P_P are converted to scores by using $-\mathrm{Log}_{10}(P_E)$ and $-\mathrm{Log}_{10}(P_P)$ respectively.

REFERENCES FOR SUPPLEMENTARY METHODS

1. Staaf J, *et al.* RNA sequencing-based single sample predictors of molecular subtype and risk of recurrence for clinical assessment of early-stage breast cancer. *NPJ Breast Cancer* **8**, 94 (2022).

2. Gendoo DM, *et al.* Genefu: an R/Bioconductor package for computation of gene expression-based signatures in breast cancer. *Bioinformatics* **32**, 1097-1099 (2016).

3. Nik-Zainal S*, et al.* Landscape of somatic mutations in 560 breast cancer whole-genome sequences. *Nature* **534**, 47-54 (2016).

4. Degasperi A*, et al.* Substitution mutational signatures in whole-genome-sequenced cancers in the UK population. *Science* **376**, (2022).

5. Davies H*, et al.* HRDetect is a predictor of BRCA1 and BRCA2 deficiency based on mutational signatures. *Nat Med* **23**, 517-525 (2017).

6. Curtis C*, et al.* The genomic and transcriptomic architecture of 2,000 breast tumours reveals novel subgroups. *Nature* **486**, 346-352 (2012).

7. Pereira B*, et al.* The somatic mutation profiles of 2,433 breast cancers refines their genomic and transcriptomic landscapes. *Nat Commun* **7**, 11479 (2016).

8. Skidmore ZL*, et al.* GenVisR: Genomic Visualizations in R. *Bioinformatics* **32**, 3012-3014 (2016).

9. Staaf J*, et al.* Whole-genome sequencing of triple-negative breast cancers in a population-based clinical study. *Nature medicine* **25**, 1526-1533 (2019).

10. Lawrence M*, et al.* Software for computing and annotating genomic ranges. *PLoS Comput Biol* **9**, e1003118 (2013).

11. Fredlund E, Staaf J, Rantala JK, Kallioniemi O, Borg A, Ringnér M. The gene expression landscape of breast cancer is shaped by tumor protein p53 status and epithelial-mesenchymal transition. *Breast Cancer Res* **14**, R113-R113 (2012).

12. Shannon P*, et al.* Cytoscape: a software environment for integrated models of biomolecular interaction networks. *Genome Res* **13**, 2498-2504 (2003).

13. Veerla S, Höglund M. Analysis of promoter regions of co-expressed genes identified by microarray analysis. *BMC Bioinformatics* **7**, 384 (2006).

14. Veerla S, Ringnér M, Höglund M. Genome-wide transcription factor binding site/promoter databases for the analysis of gene sets and co-occurrence of transcription factor binding motifs. *BMC Genomics* **11**, 145 (2010).

15. Wingender E, Dietze P, Karas H, Knüppel R. TRANSFAC: a database on transcription factors and their DNA binding sites. *Nucleic Acids Res* **24**, 238-241 (1996).

16. Sandelin A, Alkema W, Engström P, Wasserman WW, Lenhard B. JASPAR: an open-access database for eukaryotic transcription factor binding profiles. *Nucleic Acids Res* **32**, D91-94 (2004).

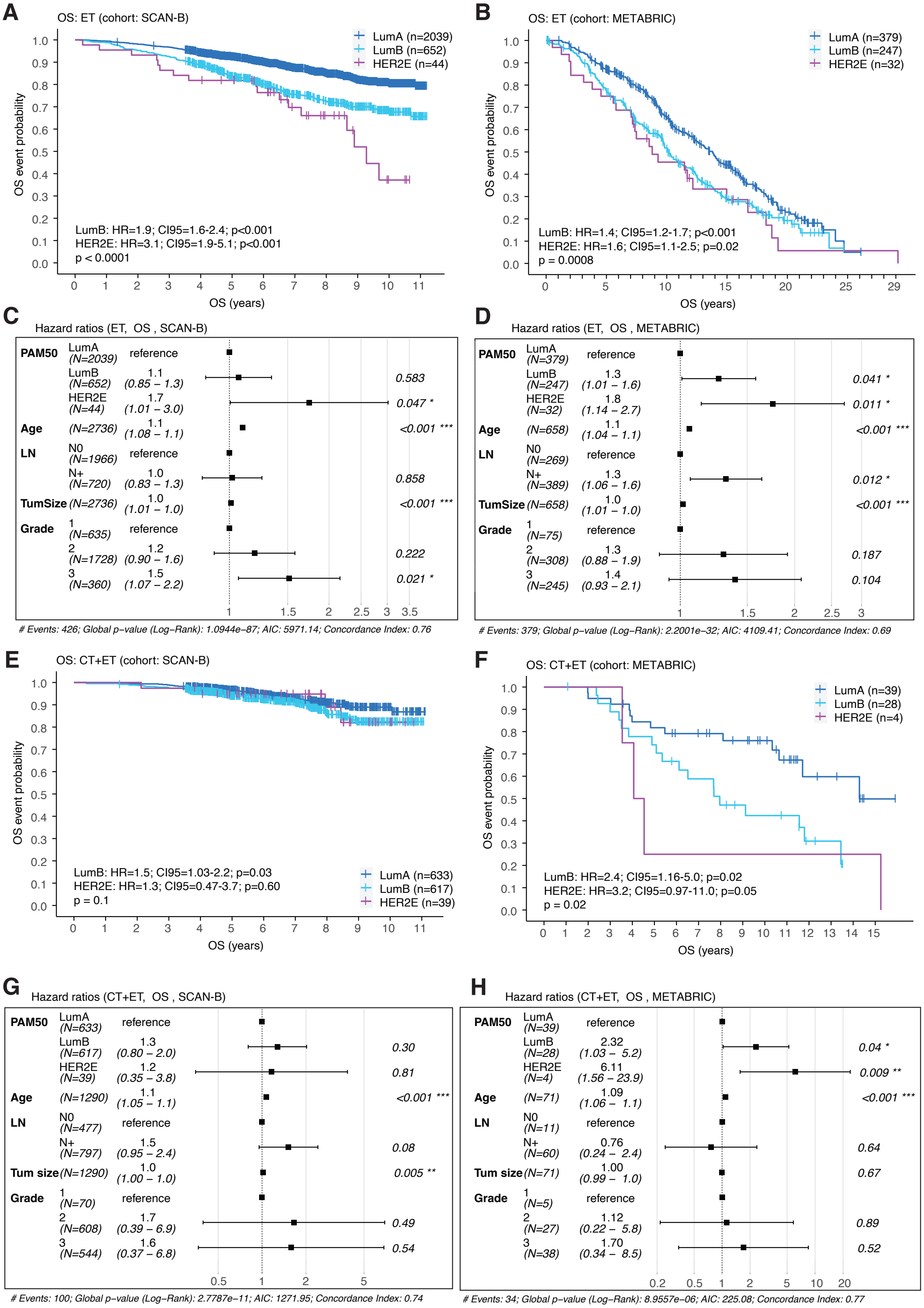

Supplementary Figure S1. Survival analyses of PAM50 subtypes in ERpHER2n tumors in SCAN-B and METABRIC using overall survival (OS) as clinical endpoint. A Kaplan-Meier analysis of SCAN-B luminal breast cancers treated with endocrine therapy (ET) including PAM50-based univariate Cox regression hazard ratios (HRs). **B** Kaplan-Meier analysis of METABRIC luminal breast cancers treated with ET including PAM50-based univariate Cox regression HRs. **C** Multivariate Cox regression HRs with 95% confidence intervals (CIs) for SCAN-B cases treated with ET. **D** Multivariate Cox regression HRs with 95% CIs for METABRIC cases treated with ET. **E** Kaplan-Meier analysis of luminal SCAN-B breast cancers that were treated with the combination of chemotherapy and endocrine therapy (CT+ET) including PAM50-based univariate Cox regression HRs. **F** Kaplan-Meier analysis of luminal METABRIC breast cancers that were treated with CT+ET including PAM50-based univariate Cox regression HRs. **G** Multivariate Cox regression hazard ratios with 95% CIs for SCAN-B cases treated with CT+ET. **H** Multivariate Cox regression hazard ratios with 95% CIs for METABRIC cases treated with CT+ET. *Differences in Kaplan-Meier survival curves were assessed by employing log-rank tests. In multivariate analyses lymph node status N0 and N+ indicate the absence or presence of regional lymph node metastases, respectively. Significance annotation: * ≤0.05; ** ≤0.01; *** ≤0.001*

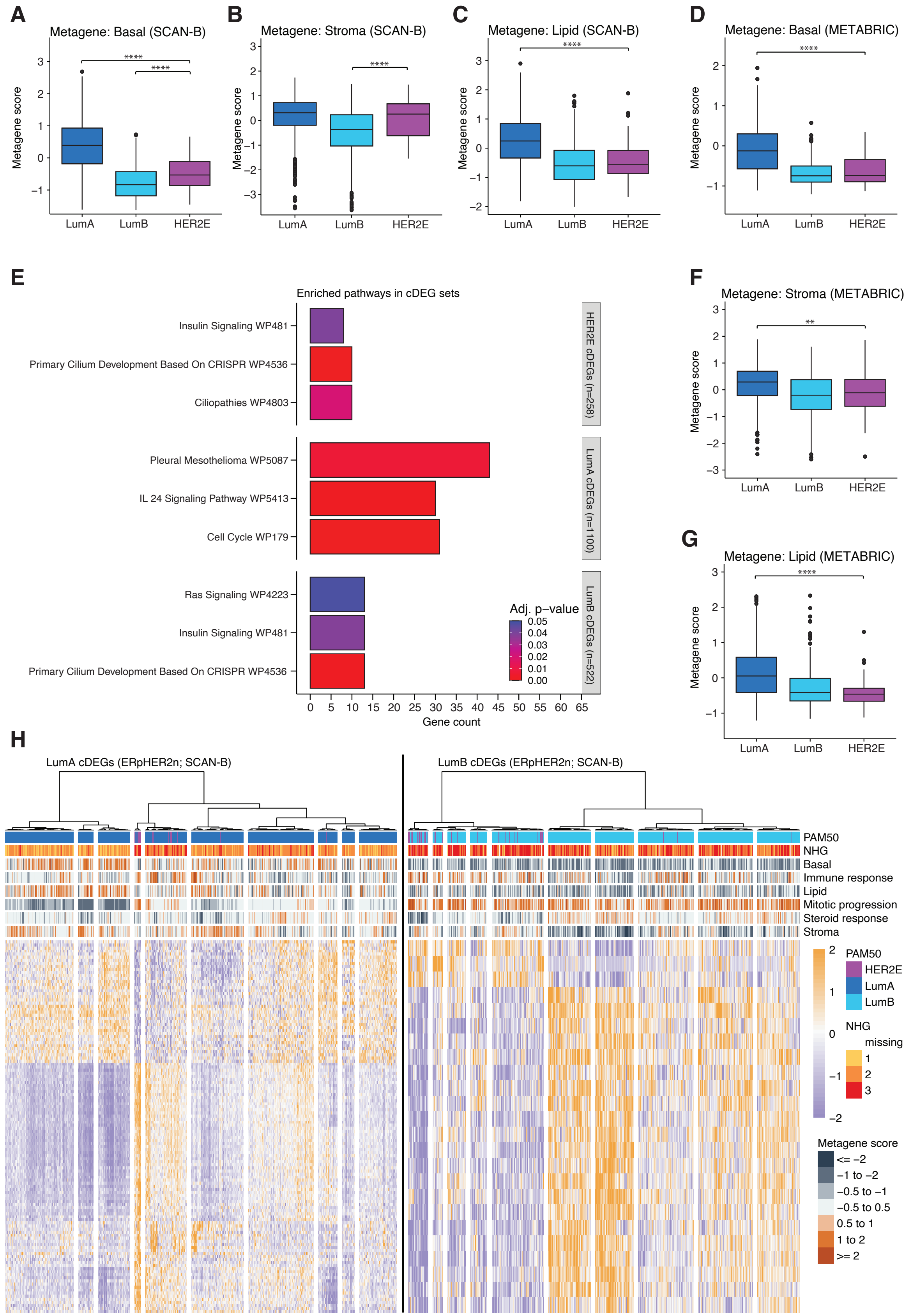

Supplementary Figure S2. Transcriptomic analyses of ERpHER2n tumors in SCAN-B and METABRIC. SCAN-B analyses were based if not otherwise illustrated on 89 HER2E tumors, 3049 LumA tumors, and 1349 LumB tumors. METABRIC analyses were based if not otherwise illustrated on 58 HER2E tumors, 601 LumA tumors, and 340 LumB tumors. **A** Basal metagene scores in SCAN-B. **B** Stroma metagene scores in SCAN-B. **C** Lipid metagene scores in SCAN-B. **D** Basal metagene scores in METABRIC. **E** Pathway enrichment results in the core HER2E-specific (HER2E cDEGs), LumA (LumA cDEGs), and LumB (cDEGs) differentially expressed gene sets based on the WikiPathways database. **F** Stroma metagene scores in METABRIC. **G** Lipid metagene scores in METABRIC. **H** Heatmaps visualizing the expression of the LumA and LumB core DEG sets (cDEGs) in SCAN-B (linkage method = "ward.D", distance method = "euclidean"). *Statistical tests: Mann-Whitney U test + FDR correction (A-D, F-G); see Enrichr (Chen et al., 2013) (E). Significance annotation: * ≤0.05; ** ≤0.01; *** ≤0.001; **** ≤0.0001. All reported p-values from statistical tests are two-sided. Boxplot elements correspond to: (i) center line = median, (ii) box limits = upper and lower quartiles, (iii) whiskers = 1.5x interquartile range.*

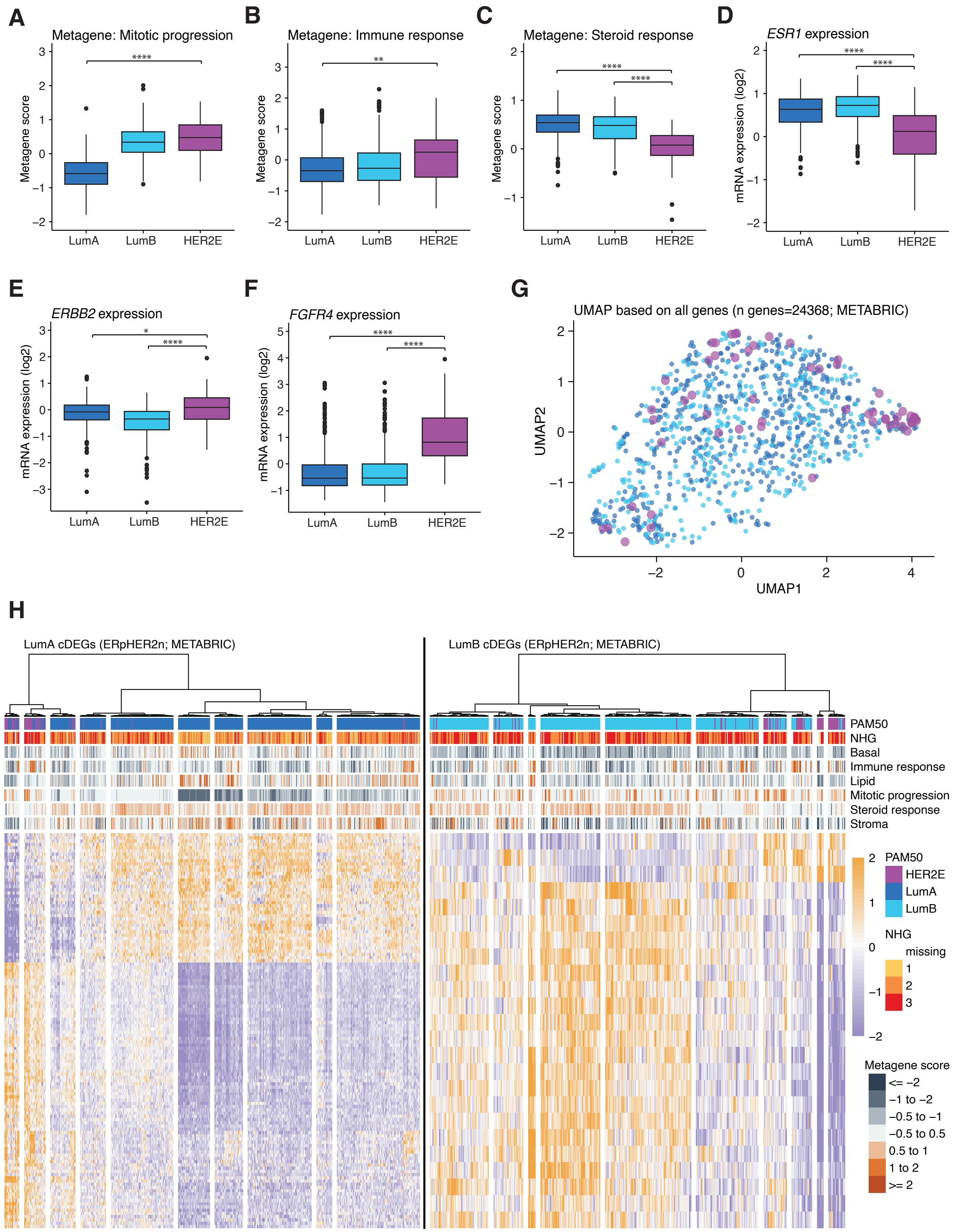

Supplementary Figure S3. Transcriptomic analyses of ERpHER2n tumors in METABRIC. METABRIC analyses were based if not otherwise illustrated on 58 HER2E tumors, 601 LumA tumors, and 340 LumB tumors. **A** Mitotic progression metagene scores. **B** Immune response metagene scores. **C** Steroid response metagene scores. **D** Scaled mRNA expression of *ESR1*. **E** Scaled mRNA expression of *ERBB2*. **F** Scaled mRNA expression of *FGFR4*. **G** UMAP analysis based on expression data of 24368 genes. Samples are colored according to subtype similar to boxplots. **H** Heatmaps visualizing the expression of the LumA and LumB core DEG sets (cDEGs) (linkage method = "ward.D", distance method = "euclidean"). *Statistical tests: Mann-Whitney U test + FDR correction (A-C); Mann-Whitney U test (D-F). Significance annotation: * ≤0.05; ** ≤0.01; *** ≤0.001; **** ≤0.0001. All reported p-values from statistical tests are two-sided. Boxplot elements correspond to: (i) center line = median, (ii) box limits = upper and lower quartiles, (iii) whiskers = 1.5x interquartile range.*

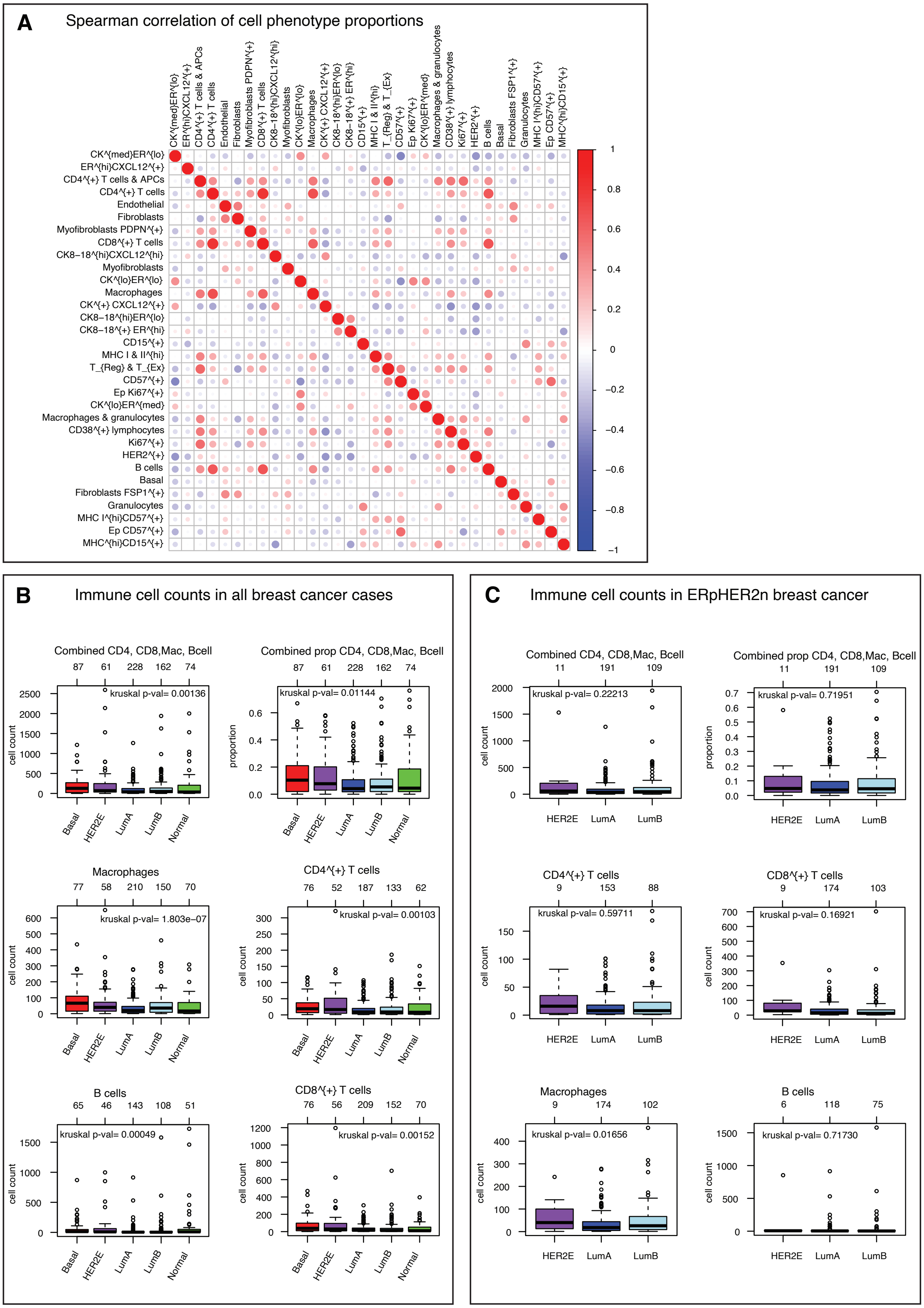

Supplementary Figure S4. Tumor infiltrating lymphocyte counts derived from single-cell METABRIC data. A Spearman correlation heatmap of all single-cell phenotype proportions as originally defined by Danenberg et al. **B** Boxplots showing the phenotype counts of CD4+ T-cells, CD8+ T-cells, Macrophages, and B-cells across PAM50 subtypes. Additionally, the figure includes boxplots of the combined counts of these immune cell types and the proportion of total cells per sample they represent in each PAM50 subtype. **C** Boxplots showing the phenotype counts of CD4+ T-cells, CD8+ T-cells, Macrophages, and B-cells across PAM50 subtypes in ERpHER2n breast cancer cases. Additionally, the figure includes boxplots of the combined counts of these immune cell types and the proportion of total cells per sample they represent in each PAM50 subtype. *Statistical significance was assessed using Kruskal-Wallis tests. All reported p-values from statistical tests are two-sided. Boxplot elements correspond to: (i) center line = median, (ii) box limits = upper and lower quartiles, (iii) whiskers = 1.5x interquartile range.*

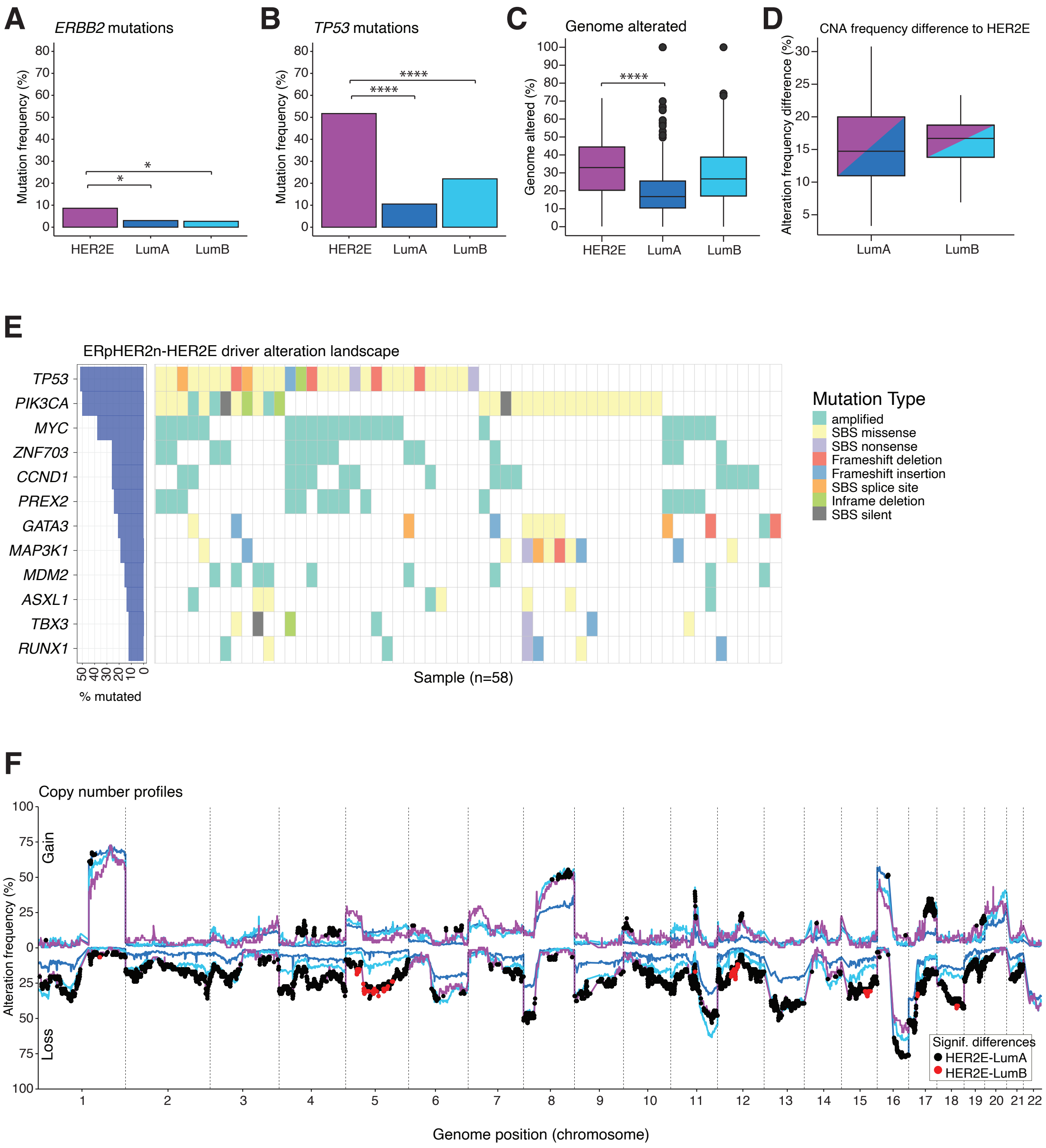

Supplementary Figure S5. PAM50 subtype associated mutational alterations and copy number patterns in ERpHER2n METABRIC tumors. METABRIC analyses were based if not otherwise illustrated on 58 HER2E tumors, 601 LumA tumors, and 340 LumB tumors. **A** *ERBB2* mutation frequencies in PAM50 subtypes. **B** *TP53* mutation frequencies in PAM50 subtypes. **C** Proportion of tumor genomes affected by copy number alterations. **D** Differences in alteration frequency of the statistically significant genes to HER2E. **E** Mutational and amplification landscape of tumor drivers in HER2E tumors. **F** Genome-wide copy number alteration profiles of gain and loss for HER2E (purple), LumA (blue), and LumB (light blue) tumors. *Statistical tests: Fisher's exact test (A-B); Mann-Whitney U test (C); Fisher's exact test + FDR correction (F). Significance annotation: * ≤0.05; ** ≤0.01; *** ≤0.001; **** ≤0.0001. All reported p-values from statistical tests are two-sided. Boxplot elements correspond to: (i) center line = median, (ii) box limits = upper and lower quartiles, (iii) whiskers = 1.5x interquartile range.*

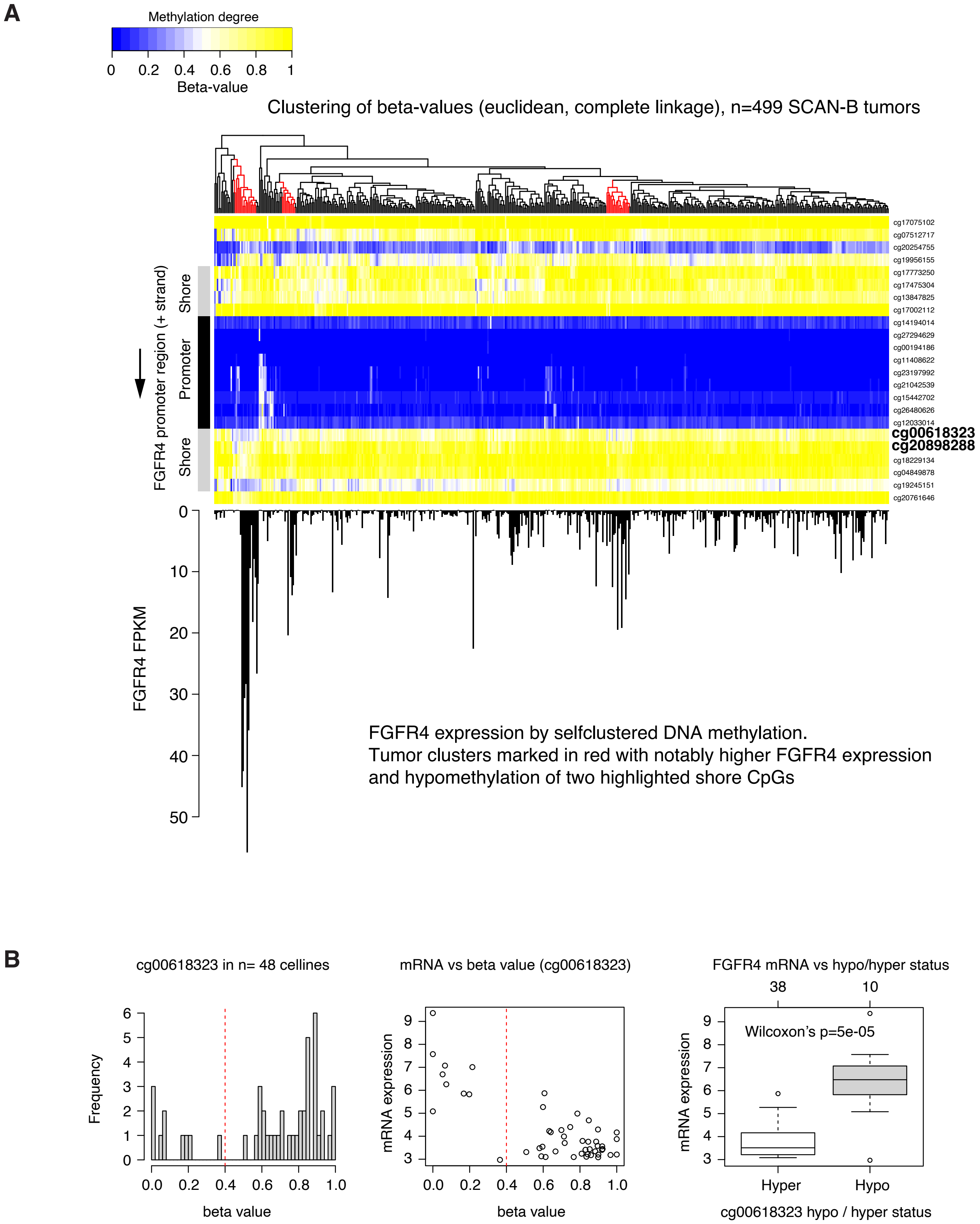

Supplementary Figure S6. DNA methylation analysis of FGFR4 promoter locus. A Heatmap of clustered beta-values for CpGs (rows) in the *FGFR4* promoter locus (defined as upstream and downstream the transcription start site) in 499 SCAN-B tumors (columns) analyzed by the Illumina EPIC v1 assay. Arrow indicates direction of expression. Lower barplot shows corresponding *FGFR4* FPKM expression values in the tumors. Clusters of tumors with marked higher FPKM expression and apparent hypomethylation of two shore CpGs highlighted in bold are labelled in red. Beta-values are not adjusted for tumor cell content, which means that the beta-value is a mix of tumor cell methylation status and normal cell (background tissue composition) methylation status. **B** Left: histogram of beta-values for the *FGFR4* shore CpG cg00618323 in 48 human breast cancer cell lines. Vertical red line corresponds to a beta-value of 0.4. Center: scatter plot of *FGFR4* gene expression versus the beta-values for cg00618323 in the cell lines. Right: gene expression versus a binary stratification into hypo (beta-value <0.4) or hypermethylation for cg00618323. *Statistical significance was assessed using Wilcoxon's test. All reported p-values from statistical tests are two-sided. Boxplot elements correspond to: (i) center line = median, (ii) box limits = upper and lower quartiles, (iii) whiskers = 1.5x interquartile range.*

Supplementary Table S1: 21-gene Recurrence Score and ROR scores and risk group classifications of SCAN-B ER+/HER2- cases.

	SCAN-B		
**Variable**	**HER2E (ref)**	**LumA**	**LumB**
**21-gene RS**	78.4 ± 24.3	23.3 ± 13.6****	55.7 ± 30.8****
**21-gene RS risk group**			
low	0 (0%)	1079 (36%)****	175 (13%)****
intermediate	3 (4%)	1058 (36%)****	165 (13%)****
High	81 (96%)	842 (28%)****	980 (74%)****
**ROR score**	73.7 ± 12.6	27.7 ± 14.3****	67.0 ± 10.8****
**ROR risk group**			
low	0 (0%)	1751 (59%)****	0 (0%)**
intermediate	7 (8%)	816 (27%)****	271 (21%)**
high	77 (92%)	412 (14%)****	1049 (79%)**

Note: Pairwise comparisons of HER2E vs. LumA and LumB, respectively. Statistics: Mann-Whitney U test for the 21-gene recurrence score (RS)/Risk of Recurrence (ROR) score and Fisher's Exact Test for 21-gene RS/ROR risk groups. Significance annotation: * ≤0.05; **≤0.01; *** ≤0.001; **** ≤0.0001. All reported p-values from statistical tests are two-sided.

Supplementary Table S2: Clinicopathological characteristics of the intrinsic luminal breast cancer subtypes in the SCAN-B review cohort.

	SCAN-B review		
**Variable**	**HER2E (ref)**	**LumA**	**LumB**
**N**	27 (3%)	508 (56%)	369 (41%)
**Patient age (years)**	62,2 ± 15,4	64,6 ± 13,1	66,7 ± 12,7
**Tumor size (mm)**	22,3 ± 9,8	17,7 ± 8,7**	22,2 ± 10,3
**PR positive**	22 (81%)	461 (91%)	301 (82%)
**HER2-low**	22 (81%)	436 (86%)	316 (86%)
**Tumor grade**			
NHG1	0 (0%)	143 (28%)****	8 (2%)
NHG2	10 (37%)	297 (59%)****	133 (36%)
NHG3	17 (63%)	67 (13%)****	225 (61%)
**LN Status**			
N0	17 (63%)	286 (56%)	196 (53%)
N+	10 (37%)	221 (44%)	173 (47%)

Note: Pairwise comparisons of HER2E vs. LumA and LumB, respectively. Percentage definitions: Across subtypes (N); within subtype (PR status, HER2-low, Tumor grade, LN status). Statistics: Fisher's exact tests for variables PR status, Grade, lymph node (LN) status, and HER2-low frequency; two-sided t-tests for variables Age and Size. Significance annotation: * ≤0.05; **≤0.01; *** ≤0.001; **** ≤0.0001. All reported p-values from statistical tests are two-sided.
