# Supplementary material for: Genomic characterization of the HER2-enriched intrinsic molecular subtype in primary ER-positive HER2-negative breast cancer
Source: Nat Commun. 2025 Mar 5;16:2208. doi: 10.1038/s41467-025-57419-z (PMC11882987; doi:10.1038/s41467-025-57419-z)
Supplement: Supplementary file 3 — Description of Additional Supplementary Files [file 41467_2025_57419_MOESM3_ESM.pdf]

Description of Additional Supplementary Files

Supplementary Data 1

Description:

Results from the differential gene expression analysis of HER2E, LumA, and LumB samples are placed in Supplementary Data 1. This includes the pairwise differential gene expression results between the groups in the SCAN-B cohort (txt table format), the pairwise differential gene expression results between the groups in the METABRIC cohort (txt table format), the defined core gene set based on overlap between SCAN-B and METABRIC results (txt table format), and gene set enrichment results (GSEA) (txt table format) for the HER2E core gene set, the LumA core gene set, and the LumB core gene set.

Supplementary Data 2

Description:

Results from the detailed analysis of the differentially expressed HER2E core gene set are placed in Supplementary Data 2. This includes the results based on gene network analysis using Cytoscape (txt table format) for the two main networks, gene set enrichment results (GSEA) for each Cytoscape gene network (txt table format), results of the differential gene expression analysis of the HER2E core gene set for ERpHER2n-HER2E tumors versus ERpHER2p tumors stratified by PAM50 subtypes (txt table format), and analyses of transcription factor binding site enrichment using both JASPAR and TRANSFAC databases of the core HER2E gene set (txt table format).

Supplementary Data 3

Description:

Results from the copy number analysis between HER2E tumors from the SCAN-B cohort versus LumA and LumB tumors from the BASIS cohort are placed in Supplementary Data 3 in the form of txt tables. This includes gene frequency estimates (percentage) for gain and loss in HER2E, LumA, and LumB tumors, Corresponding gene level Fisher's exact test p-values and adjusted p-values (FDR) for pairwise comparisons of HER2E vs LumA and LumB, and for gain and loss separately, list of genes with significant Loss in HER2E tumors versus LumA and LumB, respectively, and a list of genes with significant Gain in HER2E tumors versus LumA and LumB, respectively.

Supplementary Data 4

Description:

Whole genome sequencing somatic data for analyzed ERpHER2n HER2 tumors are placed in Supplementary Data 4 in the form of txt tables. This includes sample and QC info, HRD status based on HRDetect, exposure to mutational signatures and rearrangement signatures, called driver alterations, all detected SNVs and indels, mutation burden, kataegis status, and copy number data.

Supplementary Data 5

Description:

DNA methylation data for the FGFR4 promoter region for different cohorts are placed in Supplementary Data 5 in the form of txt tables. This includes beta values for a cohort of normal breast tissue (GSE67919), TCGA (including sample annotations), and SCAN-B tumors (including sample annotations).
